# Lag-adjusted functional network connectivity reveals sensorimotor and higher cognitive network alterations in depression

**DOI:** 10.21203/rs.3.rs-9773701/v1

**Published:** 2026-06-25

**Authors:** Malvika Sridhar, Sir-Lord Wiafe, Zening Fu, Jing Sui, Vince D. Calhoun

**Affiliations:** Center for Translational Research in Neuroimaging and Data Science (TReNDS), Georgia State University, Atlanta, GA; Center for Translational Research in Neuroimaging and Data Science (TReNDS), Georgia State University, Atlanta, GA; Center for Translational Research in Neuroimaging and Data Science (TReNDS), Georgia State University, Atlanta, GA; State Key Laboratory of Cognitive Neuroscience and Learning & IDG/McGovern Institute for Brain Research, Beijing Normal University, Beijing, China; Center for Translational Research in Neuroimaging and Data Science (TReNDS), Georgia State University, Atlanta, GA

**Keywords:** Major depressive disorder, functional network connectivity, independent component analysis, resting-state fMRI, Lag-adjusted

## Abstract

Major Depressive Disorder (MDD) involves large-scale brain network disruption at rest. Canonical zero-lag functional connectivity methods often miss temporal offsets (or “lags”) in interactions. Lag-adjusted functional connectivity captures intrinsic neural timescales (INTs) and directional signaling, offering a more sensitive framework to characterize network-level alterations. Here, we applied the NeuroMark framework to resting-state scans from 235 MDD and 284 healthy controls, identifying 105 intrinsic connectivity networks (ICNs) and their time series. To enable sub-TR estimation, time series were upsampled to 100 ms resolution. Lag-adjusted connectivity was computed as the maximal cross-correlation for each ICN pair within a ±2s window sampled at 0.1s intervals. Group differences were assessed using a regression model. Significant differences emerged between groups (p<0.05). Specifically, MDD revealed hyperconnectivity in salience–sensorimotor and sensorimotor–temporoinsular networks, alongside hypoconnectivity in salience–higher cognitive temporal and frontal networks and temporoparietal–visual systems, indicating altered coordination among sensory, emotional, and cognitive processes. An exploration of the lags revealed a non-random bias in the temporal ordering of networks operating at different INTs. This was characterized by earlier relative cortical coupling in MDD, suggesting compressed inter-network timing. These findings underscore the utility of lag-adjusted approaches for detecting impaired neural coordination, beyond alterations in connectivity strength.

## Background

The pathophysiology of major depressive disorder (MDD) is increasingly viewed as a neural circuit dysfunction associated with large-scale brain networks at rest, determined from functional magnetic resonance imaging (fMRI)^1,2^. These alterations might underpin cognitive and emotional deficits central to depressive pathology. A widely used approach in fMRI is the examination of static and dynamic resting-state functional connectivity (rsFC), which quantifies zero-lag temporal correlations between brain regions or networks at rest, either over the entire scan duration or within sliding temporal windows. This method computes Pearson correlations between blood-oxygen-level-dependent (BOLD) time series, assuming temporal synchrony, i.e., that signals from different regions activate or deactivate simultaneously without temporal shifts or delays. Meta-analytic studies of MDD using zero-lag approaches have identified hyperconnectivity of the default mode network (DMN) and hypoconnectivity of the central executive, salience network, and frontal regions^3,4,1^. However, by 2015, systematic reviews demonstrated that rsFC findings in MDD were inconsistent, small in effect size, and often statistically unreliable^5,6^. Moreover, traditional static and dynamic approaches have demonstrated low inter-scan and inter-site reliability, as well as substantial temporal variability^7,8^.

Research has shown that the brain operates across intrinsic neural timescales (INTs), whereby different networks exhibit distinct temporal dynamics; for example, sensory networks tend to show shorter timescales than higher-order networks such as the DMN^9,10^. Neural signaling is further shaped by factors such as axonal conduction delays, vascular properties, and network topology^11,12^. Consequently, if one region consistently precedes or influences another, such temporal delays are not captured by zero-lag measures and may be misinterpreted as weak or absent connectivity^13^. Lag-adjusted connectivity accounts for consistent temporal delays between regions, which can increase certain correlation strengths relative to zero-lag estimates. This re-ranking of connection strengths can determine which edges survive statistical thresholding, thereby capturing interactions that are under- or overestimated by traditional zero-lag approaches. An extension of a lag-adjusted approach marked by aberrantly early signaling of the dorsal anterior cingulate cortex (ACC) in MDD was utilized for biomarker identification^14^. Specifically, they found that 1 week of successful transcranial magnetic stimulation resulted in a statistically significant shift toward earlier activity in the dorsolateral prefrontal cortex, anterior insula, and temporoparietal junction relative to the ACC^14^.

Emerging research shows that spontaneous BOLD fluctuations correspond to low-frequency electrophysiological activity that propagates across the brain in organized spatiotemporal patterns, resulting in interregional time delays of ~1–2 seconds^15^. While low-frequency fMRI analyses have traditionally emphasized signals below 0.1 Hz, this boundary largely reflects conservative filtering choices rather than a physiological discontinuity. Independent component analysis (ICA) based decomposition enhances robustness by explicitly separating the neural signal from motion, physiological noise, and scanner drift^16^. In this context, we observe stable, reproducible network time courses with meaningful spectral content extending into the 0.1–0.15 Hz range^17^. Accordingly, we adopt a 0.15 Hz upper bound to capture a broader low-frequency regime supported by ICA-based denoising, without assuming a sharp infralow cutoff.

Intrinsic brain activity arises as a result of interactions between neural systems, which are shaped by temporally offset activation patterns described by a property known as “lag-structure”. These interactions may better reflect the complexity of psychological states underlying psychiatric disorders^14,18^. Importantly, these lag-structures are reproducible within individuals and modulated by behavioral state and pathology^15^. Most dynamic approaches are fundamentally constrained to simultaneous activation of signals, limiting their sensitivity to temporally ordered interactions between regions^19^. Instead, lag-adjusted functional connectivity methods serve as a complementary approach to traditional correlation-based analyses, offering a richer picture of INT-driven directional or sequential neural communication in disease-related disruption. We employed a lag-adjusted static rsFC to address the spatiotemporal features in neural fluctuations and characterize alterations in functional network connectivity (FNC) in MDD.

## Results

### Lag-adjusted rsFC differences

Lag-based rsFC analyses identified significant diagnostic differences between individuals with MDD and healthy controls across 17 intrinsic connectivity networks (ICN) pairs after FDR correction. These differences reflected both decreased and increased lag-adjusted functional connectivity across distributed large-scale networks.

Hypoconnectivity predominated among connections linking salience, higher-order cognitive, and temporoparietal–visual networks. The largest reduction in connectivity strength was observed between the lateral temporal pole and the right posterior insular cortex (latTP–rPIC). In contrast, hyperconnectivity was observed primarily among limbic, salience, and sensorimotor networks, with the strongest increase in connectivity between the hippocampal–entorhinal complex and the dorsal precuneus (HEC–dPRCU).

No significant clinical associations were observed between lag-adjusted connectivity strength and depression severity as measured by total HAM-D scores.

### Temporal lag structure within significant connectivity pairs

The temporal lag structure was examined within the 17 ICN pairs that showed significant lag-adjusted FNC differences. Lag offsets were referenced to the subject-level thalamo–caudate pair following the relative lag framework described by Rokham et al. due to its comparative signal stability and deep gray matter origin relative to cortical ICN pairs. No individual pairwise lag difference survived correction for multiple comparisons. However, the distribution of lag differences across pairs revealed a systematic group-level pattern.

Across the significant ICN pairs, a greater number of connections exhibited earlier lag offsets in MDD relative to healthy controls (i.e., MDD signal preceded that of controls). This directional asymmetry was statistically significant at the pattern level (binomial test, p = 0.04904), indicating a non-random bias in the relative temporal ordering of network interactions in MDD.

## Discussion

Using a lag-adjusted resting-state functional connectivity approach, this study identified 17 ICN pairs that significantly differentiated individuals with MDD from healthy controls. Importantly, these group differences reflect alterations in functional coupling strength after accounting for potential temporal misalignment between network time series, rather than differences in the magnitude or direction of delays themselves. As such, the findings highlight disruptions in inter-network coordination that persist or emerge when the strict assumption of zero-lag synchrony is relaxed. Collectively, findings indicate predominantly reduced lag-adjusted connectivity in MDD, particularly in higher-order cognitive and salience-related networks, alongside earlier relative temporal offsets in MDD compared with controls, referenced to the thalamus–caudate connection.

The strongest increase in coupling was observed between the right lentiform nucleus-right superior postcentral gyrus (rLN-rsPoCG) and the hippocampal, entorhinal complex-dorsal precuneus (HEC-dPRCU), while the strongest reduction in coupling was observed between the lateral temporal pole and the right posterior insular cortex (latTP-rPIC). The increased connectivity of the HEC and dPRCU suggests stronger coupling between episodic memory systems^20^ and self-referential, internally oriented processing^21,22^. In MDD, this may reflect excessive integration of episodic memory with internal awareness and schema, resulting in heightened self-referential thoughts and rumination. Increased connectivity between the rsPoCG and the rLN indicates strong coupling of bodily sensory processing^23^ and goal-directed motor activation in MDD^24^. The lentiform nuclei house dopaminergic neurons with strong implications in neuropsychiatric conditions. This pattern may reflect heightened salience of physical and psychomotor symptoms in depression. In contrast, decreased connectivity of the latTP and rPIC suggests weakened coordination between semantic-related emotional processing^25^ and sensorimotor processing^26^ that may indicate reduced integration of bodily sensations with higher-level emotional awareness in MDD.

The results from our lag-adjusted analyses also showed consistent patterns of increased sensorimotor (SM) connectivity, contrasting previous large-scale studies of MDD using zero-lag approaches^27,28^. This network, including the superior postcentral gyrus and the parietal and paracentral lobules, supports somatosensory processing and sensorimotor functions, with the parietal regions contributing to visuospatial processing^29^. Notably, these regions overlap substantially with nodes of the motor network examined in prior work, which reported increased thalamo- and pallido-cortical connectivity in relation to psychomotor disturbances in MDD^30,31^. Particularly, strong functional coordination was revealed between the SM and anterior insular cortices (AIC) and ACC, key components of the salience network that support stimulus detection, emotional awareness, and attentional control^32,33^. While the present study was unable to assess psychomotor symptoms directly, the observed sensorimotor hyperconnectivity may suggest involvement of motor-related thalamocortical circuits in MDD.

Another prominent pattern was widespread hypoconnectivity within and between higher-cognitive networks spanning insular–temporal, temporoparietal, and frontal subdomains. Reduced connectivity predominantly involved core hubs of the salience network, with functionally adjacent ventrolateral prefrontal cortex (associated with decision-making and emotion regulation^34,35^) and entorhinal cortex (EC) regions. These findings indicate reduced integration among networks supporting cognitive–affective, attentional control, and interoceptive processing in MDD. Hypoconnectivity was also observed in the temporoparietal junction (TPJ) and posterior temporal cortex (pTC), indicating reduced integration of regions supporting language, semantic processing, and social cognition^36,37^.

The absence of significant clinical associations with depression severity suggests that observed alterations may reflect trait-like neural markers of MDD rather than state-dependent effects. We were unable to examine relationships with specific symptom dimensions due to the lack of item-level HAM-D scores. MDD is highly heterogeneous, and prior work suggests that symptom-specific or subtype-based approaches might be more sensitive in identifying neuroimaging correlates^38–40^. These factors highlight the need for future studies using datasets with more detailed clinical characterization.

Our exploratory analysis of the temporal lag offsets among significant pairs showed that the peak occurred earlier relative to the thalamus-caudate baseline in MDD than in controls (−44 to −350 ms). This pattern suggests a compression of the normal inter-network timing structure: cortical temporoparietal, sensorimotor, and DMN-related regions in depression demonstrate earlier peak coupling relative to thalamo-striatal timing, suggesting increased temporal differentiation and reduced efficiency of cortico–subcortical coordination in depression. These findings can be interpreted within the framework of intrinsic neural timescales^9^. Sensorimotor networks tend to operate on faster timescales, whereas default-mode-related and higher-order regions integrate information more slowly^9^. Lag-based rsFC is uniquely sensitive to coordination across such heterogeneous timescales. The observed pattern of disrupted coordination between fast sensorimotor systems and slower integrative networks suggests altered large-scale temporal organization in depression. Coupling among fast-timescale systems is preserved, while coordination involving slower, integrative networks is weakened. Decreased coupling among higher-order cognitive systems in MDD, despite accounting for temporal shifts, suggests genuinely weakened top-down network communication. We also note here that these interpretations should be considered in light of the temporal resolution of BOLD signals and the indirect nature of lag estimates.

Several limitations warrant consideration. First, we were unable to distinguish between first-episode and recurrent/chronic MDD in the broader sample. Similarly, detailed medication information was unavailable across sites; only participants from site 2 were confirmed to be unmedicated first-episode MDD, possibly confounding group differences. This limited our ability to robustly account for medication effects or illness duration. Still, many MDD patients are treatment-resistant or respond variably to medication, which can make it hard to link medication class with connectivity patterns. Thus, our analysis provides valuable insight into trait-level neural changes in MDD. Second, our access to only the total HAM-D scores limited examination of associations to specific symptom dimensions (e.g., insomnia, appetite, anxiety), which may map more closely onto distinct neurobiological mechanisms. Third, our results focused on a homogeneous ethnic group, and additional analyses are needed to confirm generalizability. Finally, this analysis does not directly assess group differences in physiological lag magnitude or direction (longer, shorter, or reversed lags); rather, it assesses the relative temporal offset relative to a deep subcortical reference. Future work should examine whether timing parameters themselves carry clinical relevance. Despite these limitations, the present findings demonstrate that allowing temporal flexibility reveals meaningful connectivity differences in MDD beyond what can be captured by zero-lag approaches. This study highlights the importance of considering temporal dynamics in fMRI analyses and supports the complementary use of lag structure measures to describe the neurobiological architecture of MDD.

## Methods

### Participants

519 participants (235 MDD patients and 284 healthy control (HC) participants) were recruited from four hospital sites in China. Demographic information is provided in Table 1. All patients were diagnosed according to DSM-IV criteria based on the SCID-P interview. The current symptom severity of patients was rated using the 17-item Hamilton Depression Rating Scale (HAM-D)^41^. The study received ethical approval from the Institutional Review Board or Ethics Committee at Beijing Normal University, and informed consent was obtained from all subjects before scanning.

### MRI Acquisition

All participants were screened for MRI safety before scanning procedures. A single 8-minute resting-state scan was obtained using a 3T scanner at all four sites. A total of 240 volumes of echo-planar images were obtained (repetition time/echo time (TR/TE) = 2,000/30 ms, field of view (FOV) = 64 × 64 matrix, flip angle (FA) = 90°). Participants were instructed to keep their heads still and stay awake with their eyes closed during the scan. Memory foam and inflatable padding were used to restrict head motion.

### Preprocessing

All scans were preprocessed using SPM12 (fil.ion.ucl.ac.uk/spm/). An automated NeuroMark pipeline developed at the TReNDS Center (trendscenter.org/) was used. This included the removal of the first 5 TRs (10 seconds) to account for T1 equilibration effects, slice timing, motion correction performed using realignment, spatial normalization to the Montreal Neurological Institute (MNI) template, resliced to 3 × 3 × 3 mm isotropic voxels, and spatial smoothing (6-mm-full-width, half-maximal Gaussian kernel), and regressing non-brain tissue signals, global signal, and 24 motion parameters. The time series from each voxel was z-scored to normalize variance across the brain.

**Fig. 4.**
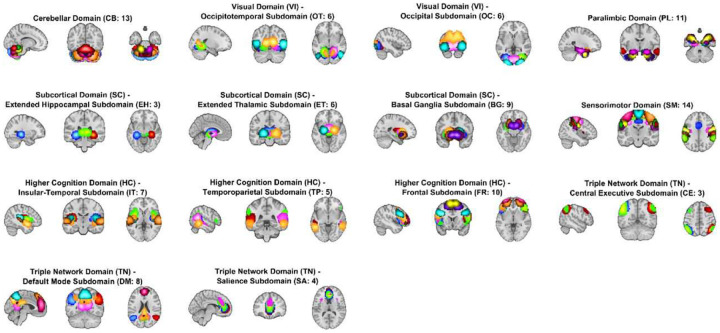
Overlayed spatial maps of the 105 intrinsic connectivity networks (ICNs) from the NeuroMark 2.2 multi-scale template are plotted above. Image from Jenson et. al. 2024 under a Creative Commons license CC BY-NC-ND 4.0^29^. CB, Cerebellar Domain; VI-OT, Visual Occipitotemporal; VI-OC, Visual Occipital; PL, Paralimbic; SC-EH, Subcortical Extended Hippocampal; SC-ET, Subcortical Extended Thalamic; SC-BG, Subcortical Basal Ganglia; SM, Sensorimotor; HC-IT, Higher Cognition Insular Temporal; HC-TP, Higher Cognition Temporoparietal; HC-FR, Higher Cognition Frontal Subdomain; TN-CE, Triple Network Central Executive; TN-DM, Triple Network Default Mode; TN-SA, Triple Network Salience.

After preprocessing, the NeuroMark framework with the functional 2.2 template was applied to identify 105 gray matter-based intrinsic connectivity networks (ICNs) and their associated time series^29^. The ICNs exhibited activation peaks distributed across whole-brain gray matter regions and were labeled based on established anatomical and functional knowledge. The 105 ICNs span 7 domains and 14 subdomains and are shown in Fig. 4. Neuromark enables comparability of networks across subjects and sessions while preserving single-scan variability in network spatial maps^42^. Prior studies have demonstrated the effectiveness of the Neuromark framework in identifying reproducible neuroimaging markers across a wide range of brain disorders^43,44^.

Each ICN time course was detrended, despiked, and bandpass-filtered to the [0.01–0.15] Hz frequency band to capture relevant neural signals as described above. These signals were then z-scored. The time courses were then interpolated by 20 using a polyphase low-pass finite impulse response (FIR) filter approach, yielding an effective temporal resolution of 100 ms^45^.

### Analysis

Lag-adjusted functional connectivity was computed on the interpolated time series for each subject by estimating, for each pair of ICA component time courses, the maximum cross-correlation within a lag window of ±2 s to account for potential hemodynamic delays. Component time series were demeaned, and the discrete cross-correlation function was computed using full convolution:

(1) $$c_{ij}(\tau )=\sum_{t}\;x_{i}(t)x_{j}(t-\tau ),$$

τ ∈ {−2: 0.1: 2} seconds, where τ denotes the lag in seconds.

**Fig 5.**
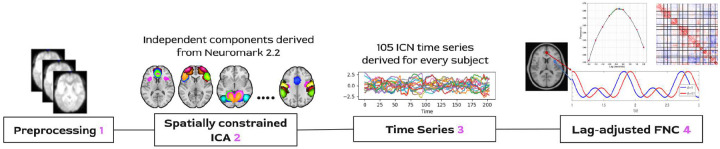
Methods Pipeline

The correlation values were evaluated within a symmetric lag window |τ|≤L, of L=2 s^18^ in increments of 100ms, corresponding to a predefined temporal range in seconds based on the effective sampling interval after upsampling. The lag corresponding to the maximal cross-correlation was identified (referred to as the “peak lag”), and this maximal correlation value was extracted as the lag-adjusted connectivity estimate for that ICN pair. A local parabolic interpolation using the correlation at this peak lag and its immediate neighbors was applied to obtain a subsampling or continuous estimate of the peak lag. The corresponding peak correlation value was normalized to yield a correlation coefficient (Figure 5). Thus, each subject yielded a single lag-adjusted FNC matrix composed of peak correlation coefficients across ICN pairs.

Subject-level lag and correlation matrices were computed independently. Pairwise lag values were referenced by subtracting the mean bilateral thalamus–caudate lag, yielding relative lag estimates. The thalamus and caudate were selected as a stable subcortical temporal anchor following prior work on hemodynamic lag estimation, which found that they form an interconnected hub with widespread cortical projections and relatively stable temporal relationships across subjects. Using this reference pair reduces ambiguity associated with global timing offsets and thus improves the interpretability of whole-brain relative lag estimates.^46,47^.

Reference-adjusted lag for every ICN pair within each subject,

(2) $$L_{\text{ref}}=\frac{L_{\text{Thal},\;\text{CaudL}}+L_{\text{Thal},\;\text{CaudR}}}{2}\;L_{ij}^{\prime }=L_{ij}-L_{\text{ref}}$$

To ensure that a single site did not drive group-level patterns, we computed site-specific group-averaged lag FNC matrices and calculated pairwise Pearson correlations (r’s) between site pairs. The MDD group showed high similarity across sites (all pairwise r > 0.91), as did the HC group (all r > 0.88), indicating strong consistency in connectivity patterns between acquisition locations.

Group comparisons on these lag matrices were conducted using a general linear model, controlling for confounding factors such as sex, age, and site, to assess whether MDD is associated with alterations in the direction or magnitude of functional connectivity. Associations of significant imaging findings with the HAM-D were explored in MDD participants. Multiple comparisons were corrected for using the false discovery rate (FDR).

## Figures and Tables

**Fig. 1.**
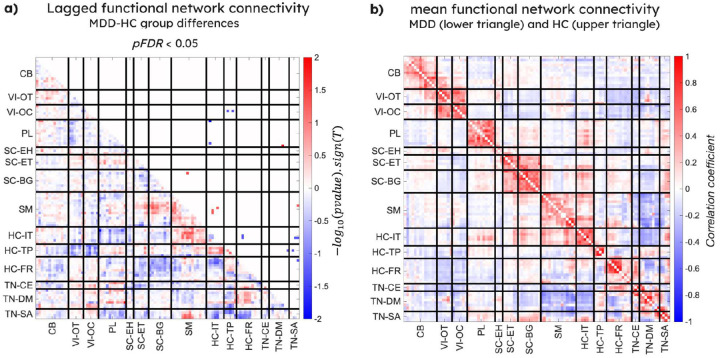

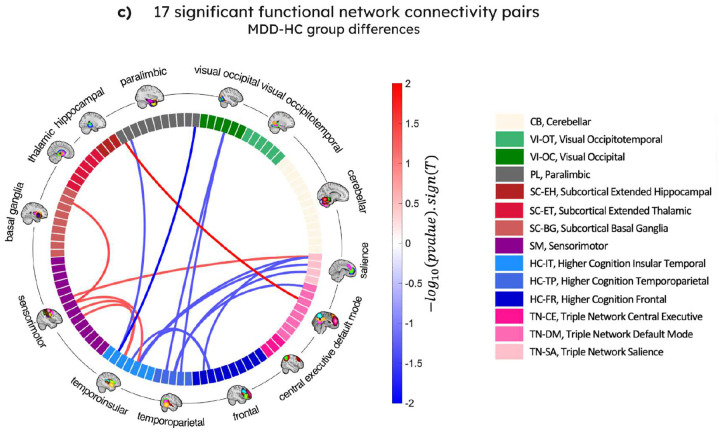
**a)** The lower triangular matrix shows FDR-corrected lagged FNC group differences (MDD–HC) for all network pairs, while the upper triangular matrix displays only the FDR-significant pairs (pFDR < 0.05). b) The lower triangular matrix shows the mean lagged FNC in MDD, while the upper triangular matrix shows the mean lagged FNC in HC. c) Circular plot thresholded to visualize the 17 significant ICN pairs. In terms of connectivity, *red=MDD>HC, blue=MDD<HC*.

**Fig. 2.**
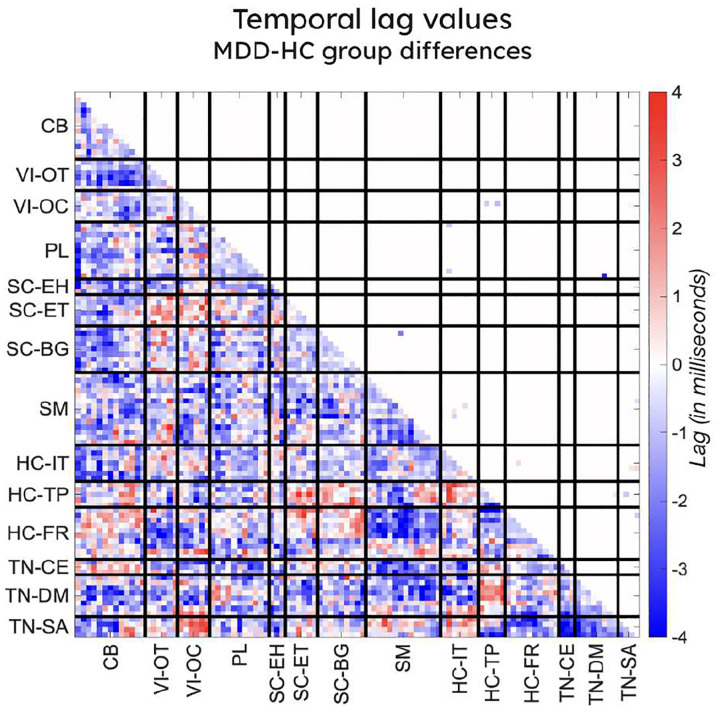
Functional network connectivity (FNC) lag matrix for all 105 pairs in the lower triangle and for the 17 pairs showing significant correlation differences in the upper triangle

**Fig. 3.**
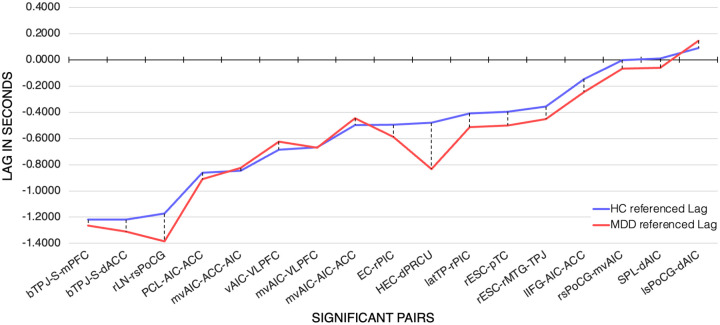
Temporal offsets of the 17 significant pairs plotted for both participant groups. Lag values are displayed in seconds and are relative to the subject-level thal-caud pair.

**Table 1.** Mean-referenced lag values for healthy controls and MDD participants, along with group differences for each significant pair.

Mean referenced lag in seconds (thal-caud)			
	HC	MDD	MDD_HC
bTPJ-S—mPFC	−1.2195	−1.2639	−0.0444
bTPJ-S—dACC	−1.2189	−1.3115	−0.0926
rLN—rsPoCG	−1.1724	−1.3860	−0.2136
PCL—AIC-ACC	−0.8600	−0.9121	−0.0520
mvAIC—ACC-AIC	−0.8473	−0.8247	0.0226
vAIC—VLPFC	−0.6857	−0.6232	0.0625
mvAIC—VLPFC	−0.6678	−0.6701	−0.0023
mvAIC—AIC-ACC	−0.4975	−0.4455	0.0521
EC—rPIC	−0.4965	−0.5886	−0.0921
HEC—dPRCU	−0.4810	−0.8325	−0.3514
latTP—rPIC	−0.4086	−0.5143	−0.1056
rESC—pTC	−0.3962	−0.5012	−0.1049
rESC—rMTG-TPJ	−0.3568	−0.4517	−0.0949
lIFG—AIC-ACC	−0.1471	−0.2446	−0.0975
rsPoCG—mvAIC	−0.0025	−0.0661	−0.0637
SPL—dAIC	0.0094	−0.0606	−0.0700
lsPoCG—dAIC	0.0909	0.1456	0.0547

ACC, anterior cingulate cortex; AIC, anterior insular cortex; DMN, default mode network; EC, entorhinal cortex; ESC, extrastriate cortex; HEC: hippocampal–entorhinal complex; IFG, inferior frontal gyrus; LN, lentiform nucleus; MTG, middle temporal gyrus; PCL, paracentral lobule; PFC, prefrontal cortex; PIC, posterior insular cortex; PoCG, postcentral gyrus; PRCU, precuneus; S, social mind areas; SPL, superior parietal lobule; TC, temporal cortex; TP, temporal pole; TPJ, temporoparietal junction; VLPFC, ventrolateral prefrontal cortex. Lowercase prefixes indicate laterality or subregional divisions (b, bilateral; d, dorsal; l, left; lat, lateral; m, medial; mv, medial ventral; p, posterior; r, right; s, superior; v, ventral)

**Table 2.** Demographics Information.

	MDD	HC
Sample	235	284
Age (mean ± SD)	32.29 ±10.89	31.32 ± 10.69
Sex (M/F)	93: 142	104: 180
HAM-D (mean ± SD)	20.49 ± 6.15	N/A

F, female; HAM-D, Hamilton depression rating scale; HC, healthy controls; M, male; MDD, major depressive disorder; SD, standard deviation

**Table 3.** Site-specific acquisition information.

Site	Sample		Scanner	Head coil	Axial slice acquisition		
	MDD	HC			Count	Thickness	Gap
1	71	71	3T Siemens	12-channel phased-array	33	4 mm	0.6 mm
2	78	107	3T Philips	8-channel phased-array	38	4 mm	0 mm
3	63	70	3T Siemens	32-channel phased-array	38	4 mm	0.7 mm
4	29	30	3T Siemens	12-channel phased-array	38	4 mm	0 mm

F, female; HC, healthy controls; MDD, major depressive disorder; T, tesla.

## Data Availability

Contact information and resources for obtaining further details for the private dataset utilized in the present study are as follows: Vince D. Calhoun (vcalhoun@gsu.edu), Tri-Institutional Center for Translational Research in Neuroimaging and Data Science (TReNDS), Atlanta, GA. Jing Sui (18311339660@163.com), State Key Laboratory of Cognitive Neuroscience and Learning & IDG/McGovern Institute for Brain Research, Beijing Normal University, Beijing, China
